# The Vasorelaxant Effect of *p*-Cymene in Rat Aorta Involves Potassium Channels

**DOI:** 10.1155/2015/458080

**Published:** 2015-01-15

**Authors:** Martapolyana T. M. Silva, Fernanda P. R. A. Ribeiro, Maria Alice M. B. Medeiros, Pedrita A. Sampaio, Yonara M. S. Silva, Morganna T. A. Silva, Jullyana S. S. Quintans, Lucindo J. Quintans-Júnior, Luciano A. A. Ribeiro

**Affiliations:** ^1^Programa de Pós-Graduação em Recursos Naturais do Semiárido (PGRNSA), Universidade Federal do Vale do São Francisco (UNIVASF), Campus Centro, 56304-205 Petrolina, PE, Brazil; ^2^Curso de Farmácia, Universidade Federal do Vale do São Francisco (UNIVASF), Campus Centro, 56304-205 Petrolina, PE, Brazil; ^3^Centro de Ciências Biológicas e da Saúde, Departamento de Fisiologia, Universidade Federal de Sergipe, Campus de São Cristóvão, 49100-001 Aracaju, SE, Brazil; ^4^Colegiado de Ciências Farmacêuticas (CFARM), Universidade Federal do Vale do São Francisco (UNIVASF), Campus Centro, 56304-205 Petrolina, PE, Brazil

## Abstract

The monoterpenes are the main constituents of most essential oils and *p*-cymene is a monoterpene commonly found in various species of aromatic herbs, which has been reported for anti-inflammatory, antinociceptive, and antimicrobial activities. However, there is no report concerning its pharmacological activity on the vascular smooth muscle. The aim of current work was to investigate the effects of *p*-cymene in isolated rat aorta and also study its mechanism of action. In this work, we show that *p*-cymene has a relaxant effect, in a dose-dependent way, on the vascular smooth muscle, regardless of the presence of the endothelium. Using a nonselective potassium channel blocker, the CsCl, the relaxant effect of *p*-cymene was attenuated. In the presence of more selective potassium channels blockers, such as TEA or 4-AP, no change in the relaxant effect of *p*-cymene was evidenced, indicating that BK_Ca_ and K_V_ channels are not involved in that relaxant effect. However, in the presence of glibenclamide or BaCl_2_, K_ATP_ and K_ir_ blockers, respectively, the relaxant effect of *p*-cymene was attenuated. The data presented indicate that *p*-cymene has a relaxing effect on rat aorta, regardless of the endothelium, but with the participation of the K_ATP_ and K_ir_ channels.

## 1. Introduction

Essential oils are volatile, have strong smell, present a complex composition, and are formed by the secondary metabolism of aromatic plants [1]. Due to their characteristic odor, essential oils are widely used in fragrances, cosmetics, and sanitary products. Besides, they are used in holistic medicine therapies such as aromatherapy. Furthermore, due to the complexity of their constituents, these oils are used in medicines, in dentistry, in pest control, and in canned food preservatives [2].

Some studies indicate that many of these essential oils have biological activities, such as cardiovascular effects [3], inhibition of the oxidation of LDL cholesterol, and blood pressure lowering effect [4, 5].

It is known that the major chemical components of the essential oils are monoterpenes and sesquiterpenes [6]. A monoterpene that is commonly found in various species of herbs is the* p*-cymene. Indeed, this monoterpene is present in the volatile oils of over 100 plant species and occurs naturally in more than 200 kinds of foods, such as orange juice, grapefruit, tangerine fruit, carrots, raspberries, butter, nutmeg, oregano, and most spices [7].

It has been demonstrated that the* p*-cymene has anti-inflammatory, analgesic, and antinociceptive activities [8–10]; immunomodulatory effect [11]; antibacterial activity against* Escherichia coli* [12]; hypotension and bradycardia effects in urethane anaesthetized rats [13]; and antioxidant activity and relaxant effect in rat aorta [14]. Although there are reports on the relaxing activity in rat aorta, little is known about the mechanism of action of this monoterpene regarding its relaxing effect.

Concerning the aforementioned, the objective of this work was to access the role of endothelium and potassium channels in the relaxing effect of* p*-cymene in isolated rat aorta.

## 2. Materials and Methods

### 2.1. Animals and Ethics Considerations

In all experiments, male Wistar rats were used, weighing 250–350 g, fed on standard rat chow with free access to food (PURINA, Brazil) and tap water* ad libitum*. The animals were maintained in a 12 h light–dark cycle (lights on: 06:00–18:00 h) under controlled temperature conditions (21 ± 1°C). All experimental procedures were performed in accordance with the guidelines proofed by the Ethics Committee on Animal Experimentation of UNIVASF (CEUA/UNIVASF number 0002/131211) and follow the recommendations of the National Council for Control of Animal Experimentation of Brazil (CONCEA).

### 2.2. Chemicals and Krebs Solution

**Figure 1:**
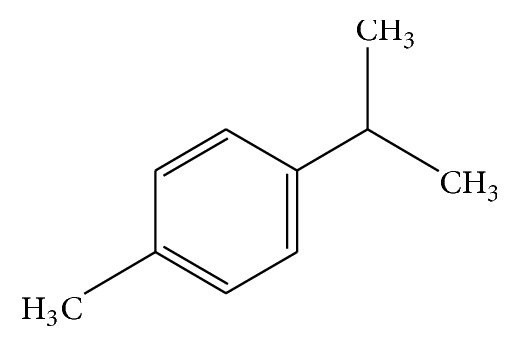
Chemical structure of* p*-cymene.

For chemicals, acetylcholine chloride (ACh), cesium chloride (CsCl),* p*-cymene (Figure 1), glibenclamide, dimethyl sulphoxide (DMSO), phenylephrine hydrochloride (PE), tetraethylammonium chloride (TEA), 4-aminopyridine (4-AP), and barium chloride (BaCl_2_) were purchased from Sigma-Aldrich (St. Louis, MO, USA). All salts used in Krebs solution were purchased from Vetec QuímicaFina Ltda. (Duque de Caxias, RJ, Brazil).* p*-Cymene was dissolved in DMSO (100%) and diluted in distilled water according to the requirements of each experiment.

For all experiments with isolated rat aorta, Krebs salt solution was used, with the composition and concentration (in mM) as follows: NaCl (118), KCl (4.6), MgSO_4_·7H_2_O (5.7), NaH_2_PO_4_·H_2_O (1.1), CaCl_2_ (2.5), NaHCO_3_ (25), and glucose (11) with pH adjusted to 7.4 with 1 N HCl solution.

### 2.3. Preparation and Protocols of Isolated Rat Aorta

In order to record the isotonic contractions, isolated rat aorta rings (4-5 mm) were suspended in organ chambers (10 mL) of an isolated organ bath system, model EFF 321 from Insight Instruments (São Paulo, SP, Brazil), and attached to isometric transducers, model TRO015 from Panlab S.L.U. (Barcelona, Spain), coupled to a bridge system amplifier 321 from Insight Instruments (São Paulo, SP, Brazil) and connected to a computer.

The rats were euthanized following the principles of Laboratory Animal Care in accordance with the guidelines of bioethics committee. The thoracic aorta was removed from the animals, immediately immersed in Krebs solution bubbled with carbogen mixture (95% O_2_ plus 5% CO_2_), and cleaned up from fat and connective tissues. The aortas were cut into 5 mm long rings and transferred to an organ chamber with Krebs solution at 37°C. The aortic rings were allowed to stabilize for 60 min at preload tension of 1 g (baseline). During the resting time, the Krebs solution was changed every 15 min to avoid accumulation of metabolites. After that elapsed time, the aortic rings were contracted by the addition of PE (1 *μ*M) and the isometric tension was recorded. When a stable contraction was attained (plateau reached in 15–20 min), ACh (1 *μ*M) was added to the organ bath to confirm the presence or absence of functional endothelium, as described earlier by Furchgott and Zawadzki [15]. Aortic rings without endothelium were obtained by means of the mechanical removal of the endothelial layer. After 30 min, a second PE-induced stable contraction was induced and cumulative-concentration curves by* p*-cymene were performed in absence or in the presence of functional endothelium, CsCl (5 mM), TEA (1 mM or 10 mM), 4-AP (3 mM), BaCl_2_(0.1 mM), or glibenclamide (3 *μ*M) for the assessment of the participation of potassium channels in the relaxant effect of* p*-cymene. In experiments involving the use of potassium channel blockers, the absence of the endothelium was verified by the lack of relaxation induced by Ach. All potassium channels blockers were added 30 min before a second contraction induced by phenylephrine.

### 2.4. Statistics

All the data are expressed as the mean ± S.E.M. and *n* refers to the number of animals used in each set of experiments. The EC_50_ values (half-maximal effective concentration) were calculated through nonlinear regression of the concentration-response curves of* p*-cymene in each protocol. The *E*_max⁡_ value refers to a maximal effect induced by a substance in percentage, being equal to 0% in maximum contraction tension elicited by phenylephrine and 100% when initial preload tension level was reached (baseline). Differences between the means were statistically compared using nonpaired Student's *t*-test, where such differences were considered significant when *P* values were less than 0.05. Statistical analyses were performed using the software Graph-Pad Prism^©^ 5.1 (GraphPad Software Inc., San Diego, USA).

## 3. Results

### 3.1. Effect of* p*-Cymene in Aortic Rings with and without Endothelium

**Figure 2:**
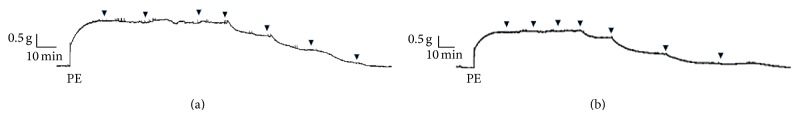
Representative original record of the effect of* p*-cymene on the isolated rat aortic rings contracted by phenylephrine with (a) and without (b) endothelium. The arrows represent the time-course of the* p*-cymene administration (10^−6^ to 10^−3^ M).

**Figure 3:**
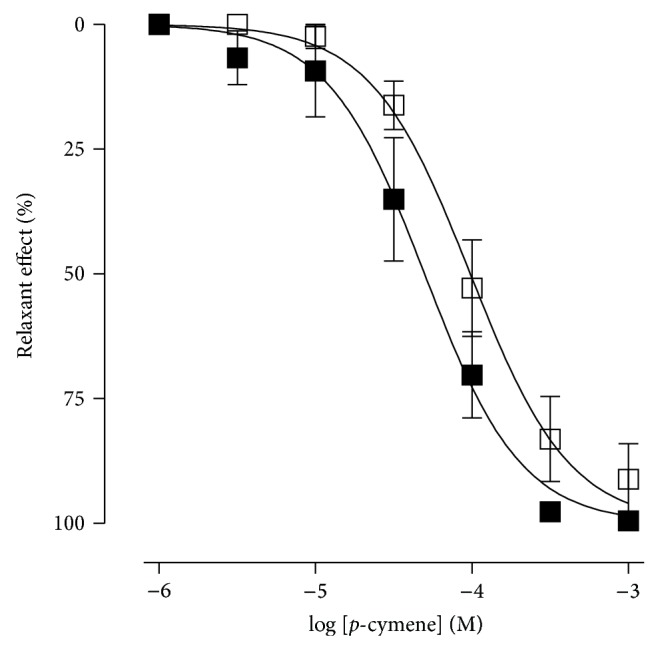
Relaxant effect of* p*-cymene on the isolated rat aortic rings contracted by phenylephrine with (■) and without (□) endothelium.

The* p*-cymene relaxed the isolated rat aorta rings, precontracted by phenylephrine, in a concentration-dependent manner, in the presence and absence of the endothelium (Figure 2), reaching an *E*_max⁡_ value of 100% of relaxation at the concentration of 10^−3 ^M (Figure 2). For rings with endothelium, the* p*-cymene presented an EC_50_ value of 5.8 ± 1.6 × 10^−5^ M and for rings without endothelium, an EC_50_ value of 1.9 ± 0.9 × 10^−4^ M (Figure 3). There was no statistical difference between values of *E*_max⁡_ and EC_50_ presented by* p*-cymene in the presence and absence of the endothelium. The contractile effect of phenylephrine was completely reestablished after 30 min of washout of the organ with a fresh new Krebs solution, to remove the* p*-cymene of the organ chambers (data not shown). Concerning a possible direct effect of the solvent, the addition of DMSO (1% v/v), which was the maximum concentration of the solvent in the organ bath in our experiments, caused no significant effect on the tonus of the aortic rings constricted (data not shown).

### 3.2. Effect of* p*-Cymene in the Presence or Absence of CsCl

**Figure 4:**
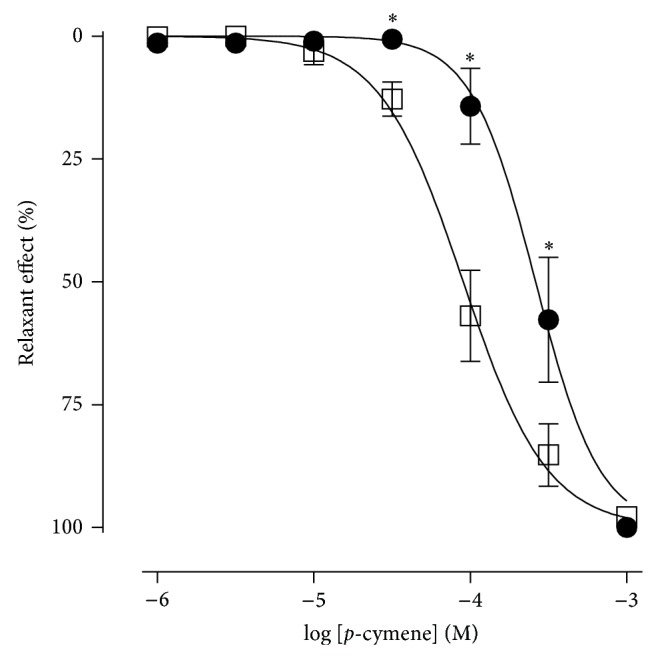
Relaxant effect of* p*-cymene on the isolated rat aortic rings without endothelium, contracted by phenylephrine, in the absence (□) and presence of CsCl 5 mM (●). ^*^ *P* < 0.05 (unpaired *t*-test: absence × presence of CsCl).

In the presence of CsCl,* p*-cymene also relaxed the aortic rings without endothelium in a concentration-dependent manner. However, the dose-response curve was shifted to the right when compared to the control curve (Figure 4). Nevertheless, no change was observed in *E*_max⁡_, which was reached at a concentration of 10^−3^ M, similarly to the control. The EC_50_ values were 1.1 ± 0.2 × 10^−4^ M and 2.4 ± 0.4 × 10^−4^ M for the control condition and in the presence of CsCl, respectively, showing statistical difference between them.

### 3.3. Effect of* p*-Cymene in the Presence or Absence of TEA or 4-AP

**Figure 5:**
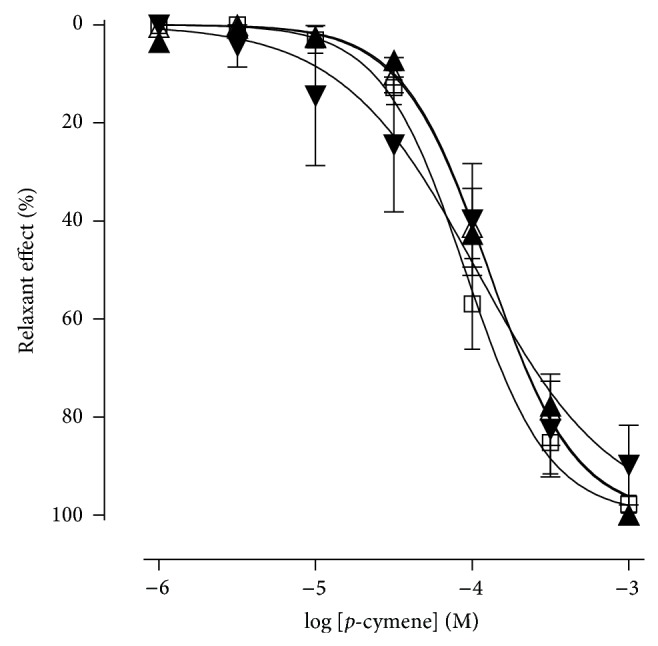
Relaxant effect of* p*-cymene on the isolated rat aortic rings without endothelium, contracted by phenylephrine, in the absence (□) and presence of TEA 1 mM (▲), TEA 10 mM (△), or 4-AP 3 mM (▼).

The* p*-cymene relaxed the aortic rings without endothelium, in a concentration-dependent manner, in both the absence (control) and the presence of TEA (1 mM), TEA (10 mM), or 4-AP (3 mM), with EC_50_ values of 1.1 ± 0.3 × 10^−4^ M, 1.6 ± 0.5 × 10^−4^ M, 1.3 ± 0.2 × 10^−4^ M, and 2.2 ± 0.1 × 10^−4^ M, respectively, showing no statistical differences between them (*P* < 0.05). In all experimental conditions, the *E*_max⁡_ value was 100% and reached 10^−3^ M (Figure 5).

### 3.4. Effect of* p*-Cymene in the Presence or Absence of BaCl_2_ or Glibenclamide

**Figure 6:**
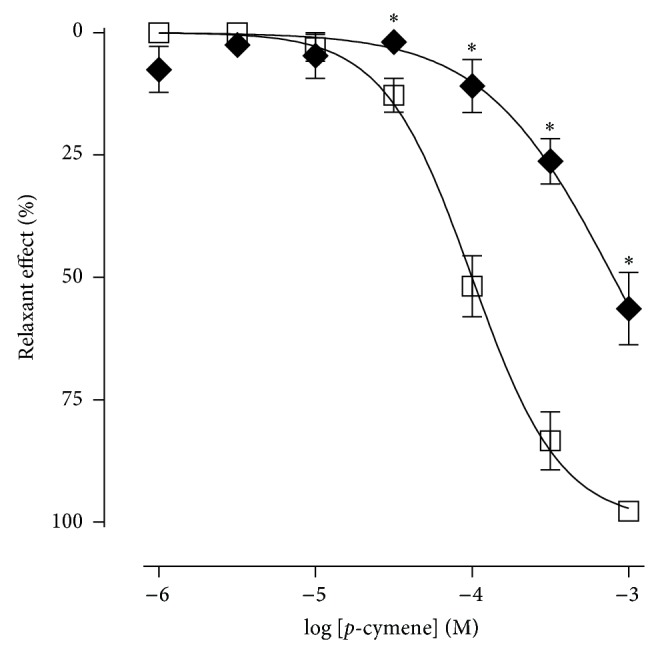
Relaxant effect of* p*-cymene on the isolated rat aortic rings without endothelium, contracted by phenylephrine, in the absence (□) and presence of BaCl_2_ 0.1 mM (◆).^*^ *P* < 0.05 (unpaired *t*-test: absence × presence of BaCl_2_).

The* p*-cymene relaxed the aortic rings without endothelium, in a concentration-dependent manner, in both the absence (control) and the presence of BaCl_2_. However, in the presence of BaCl_2_, the dose-response curve was significantly shifted to the right with a change in *E*_max⁡_ when compared to the control curve (Figure 6). The presence of BaCl_2_ reduced the *E*_max⁡_ from 97.8 ± 1.7% to 56.4 ± 7.4% and increased the EC_50_ value from 1.1 ± 0.2 × 10^−4^ M to 8.8 ± 1.5 × 10^−4^ M. Both values present a statistical difference between control values.

**Figure 7:**
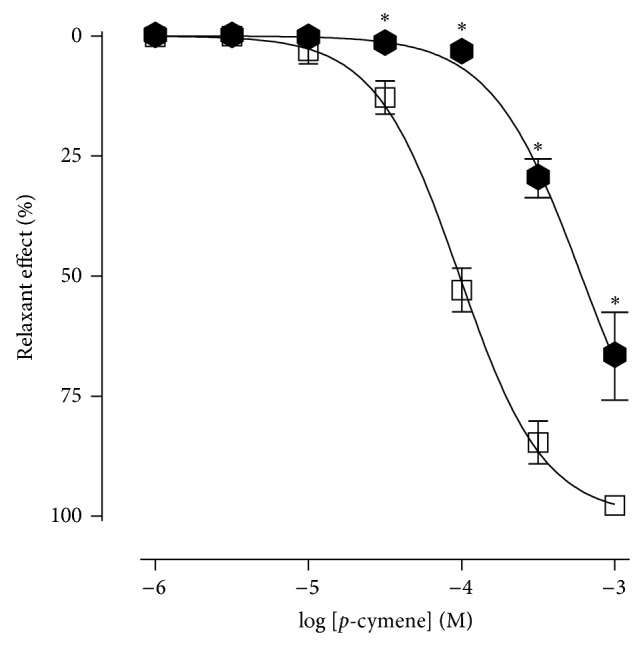
Relaxant effect of* p*-cymene on the isolated rat aortic rings without endothelium, contracted by phenylephrine, in the absence (□) and presence of glibenclamide 3 *μ*M (*⬢*).^*^ *P* < 0.05 (unpaired *t*-test: absence × presence of glibenclamide).

In the same way,* p*-cymene relaxed the aortic rings without endothelium in both the absence (control) and the presence of glibenclamide, but in the presence of that potassium channel blocker, the dose-response curve was significantly shifted to the right with a decrease in *E*_max⁡_ value when compared to the control curve (Figure 7). The presence of glibenclamide reduced the *E*_max⁡_ from 98.0 ± 1.1% to 67.4 ± 9.1% and increased the EC_50_ value from 1.1 ± 0.3 × 10^−4^ M to 6.8 ± 1.2 × 10^−4^ M. Both values present a statistical difference between control values.

## 4. Discussion

In this work, we demonstrated that* p*-cymene has a dose-dependent vasorelaxant effect, which was independent of the vascular endothelium with a marked involvement of potassium channels, especially the K_IR_ and K_ATP_ channels.

Smooth muscle contraction is ultimately determined by phosphorylation of the 20 kDa myosin light chain subunits (MLC_20_), which can occur through the Ca^2+^/calmodulin-dependent actions of myosin light chain kinase (MLCK) or through the Ca^2+^-independent actions of many additional kinases. The main event that determines the activation of that Ca^2+^/calmodulin pathway with a subsequent activation of MLCK, which leads to the contraction of the smooth muscle, is increased intracellular Ca^2+^ [16]. Two classical ways to increase the Ca^2+^ driving to muscle contraction are pharmacomechanical and electromechanical coupling mechanisms. The first mechanism involves Ca^2+^ mobilization of sarcoplasmic reticulum via G-protein-coupled receptors as well as Ca^2+^ entry by Ca_V1.2_ calcium channels; the second mechanism involves change in membrane potential and activation of Ca_V1.2_ channels, with a subsequent Ca^2+^ influx from extracellular medium and increase in its intracellular levels [17].

On the other hand, the mechanisms that lead to relaxation may involve multiple signaling pathways. In the vascular smooth muscle cells, both endothelium-derived factors and the membrane potential are important in the regulation of the vascular tone [18]. Thus, a possible role for the endothelium and K^+^ channels in the relaxation induced by* p*-cymene was investigated in this work.

The endothelium produces and releases at least three important relaxant factors: nitric oxide (NO), prostacyclin (PGI_2_), and the endothelium-derived hyperpolarizing factor (EDHF) [15, 19, 20]. Currently, it is generally accepted that endothelium-derived factors, such as NO, have an important role in modulating relaxation in the rat aorta. However, our results showed that* p*-cymene relaxes aortic rings in an endothelium-independent manner, with similar *E*_max⁡_ and EC_50_ values in the presence or the absence of endothelium. That suggests that the effect of* p*-cymene does not depend on the release of these endothelium-derived relaxant factors.

Since the Ca_V1.2_ is the main type of voltage-opened calcium channel present in smooth muscle cells [21], which is important for the development and maintenance of contraction in smooth muscle, it is known that the opening and closing of Ca_V1.2_ are closely related variations in membrane potential. Therefore, potassium channels play an important role in controlling this membrane potential and consequently control the function of Ca_V1.2_ channels [22]. Once determined that the relaxing effect of* p*-cymene is not dependent on the presence of the vascular endothelium, denoting a likely direct effect on the smooth muscle, investigation of the role of potassium channels in that effect was carried out in the absence of endothelium. In this work it was shown that, in the presence of CsCl, a well-known blocker of potassium channels [23], the vasorelaxant effect of* p*-cymene was attenuated, indicating that some types of potassium channels may be involved in its relaxant effect.

Several different types of potassium channels are present in the vascular smooth muscle, including the calcium-activated potassium (K_Ca1.1_ or BK_Ca_), blocked by TEA 1–10 mM [22], voltage-gated potassium channels (K_V_), blocked by 4-AP [23], inward rectifying potassium channel (K_ir_), blocked by Ba^2+^ [24], and ATP-sensitive potassium channels (K_ATP_), blocked by glibenclamide [25].

In this work, it was demonstrated that* p*-cymene relaxed aortic rings in the presence of TEA 1 or 10 mM, which in the lowest concentration is a selective BK_Ca_ blocker and in higher concentration is a nonselective blocker for some types of potassium channels, as well as in the presence of K_V_ blocker, the 4-AP, without significant differences in its *E*_max⁡_ and EC_50_ values. These data indicate that neither BK_Ca_ nor K_V_ would be involved in the relaxing effect of* p*-cymene. However, in the presence of both Ba^2+^ and glibenclamide, the vasorelaxant response to* p*-cymene was significantly reduced, with changes in the *E*_max⁡_ and EC_50_, indicating that K_ir_ and K_ATP_ could be involved in its relaxing effect on aortic rings.

*p*-Cymene is a known monoterpene, which has some applications in the odorant industry and also many biological effects, such as the modulation of MAPK and NF-kappa B activation [11], anti-inflammatory and antinociceptive effects [9], and antimicrobial effects in food [26]. Here we demonstrate, for the first time in literature, the* p*-cymene effect on the vascular smooth muscle, trying to determine its relaxing mechanism of action. Despite our best efforts, further studies are necessary for a better comprehension of its effect on the smooth muscle.
